# Infection prevention guidelines and considerations for paediatric risk groups when reopening primary schools during COVID-19 pandemic, Norway, April 2020

**DOI:** 10.2807/1560-7917.ES.2020.25.22.2000921

**Published:** 2020-06-04

**Authors:** Tone Bjordal Johansen, Elisabeth Astrup, Solveig Jore, Hege Nilssen, Bente Barton Dahlberg, Claus Klingenberg, Are Stuwitz Berg, Margrethe Greve-Isdahl

**Affiliations:** 1 The Norwegian Institute of Public Health, Oslo, Norway; 2 European Public Health Microbiology Training Programme (EUPHEM), European Centre for Disease Prevention and Control (ECDC), Stockholm, Sweden; 3 The Norwegian Directorate for Education and Training, Oslo, Norway; 4 Paediatric Research Group, Faculty of Health Sciences, University of Tromsø-Arctic University of Norway, Tromsø, Norway; 5 Department of Paediatrics and Adolescence Medicine, University Hospital of North Norway, Tromsø, Norway

**Keywords:** COVID-19, school closure, transmission of COVID-19 in children, paediatric risk groups, infection prevention and control guidelines, primary schools

## Abstract

In response to the coronavirus disease (COVID-19) pandemic, most countries implemented school closures. In Norway, schools closed on 13 March 2020. The evidence of effect on disease transmission was limited, while negative consequences were evident. Before reopening, risk-assessment for paediatric risk groups was performed, concluding that most children can attend school with few conditions requiring preventative homeschooling. We here present infection prevention and control guidelines for primary schools and recommendations for paediatric risk groups.

In response to the coronavirus disease (COVID-19) pandemic, 185 countries had implemented regional or national school closures by 1 April 2020, affecting 89.4% of the world’s children [1]. We here present guidelines developed for the reopening of primary schools in Norway.

## COVID-19 epidemic in Norway

Norway reported its first COVID-19 case on 26 February 2020. Quarantine and isolation were implemented for travellers coming to Norway from affected areas and for confirmed COVID-19 cases on 7 March, effective retroactively from 22 February. On 12 March, the government announced a series of restrictive infection control measures after a rapid increase in cases and evidence of community transmission (Figure). These included border control and a travel ban; closure of daycares, schools, universities and businesses; and a ban on mass gatherings. A strict lockdown was never imposed, but the general rule was to work from home and avoid public transportation. The population mobility dropped dramatically overnight [2,3].

**Figure:**
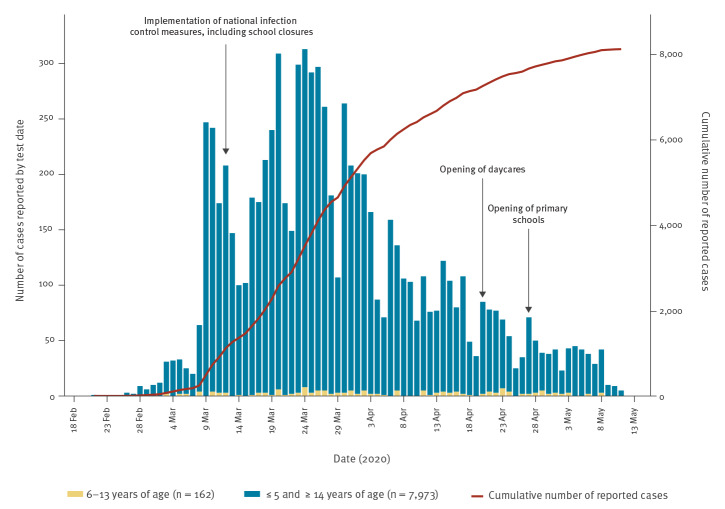
Number of confirmed COVID-19 cases reported to the Norwegian Surveillance System for Communicable Diseases (MSIS), Norway, 21 February–11 May 2020 (n = 8,135)

The figure illustrates number of confirmed cases reported according to test date among children 6 to 13 years of age and in the population ≤ 5 and ≥ 14 years of age, as well as the cumulative number of reported cases. Dates for implementation of infection control measures, including school closures, are illustrated.

On 24 March 2020, the Norwegian Institute of Public Health (NIPH) presented a risk assessment with multiple scenarios based on different target effective reproduction numbers (R*_eff_*). The government decided to follow an aggressive strategy aiming for a R*_eff_* < 1 to avoid overwhelming the healthcare system [2]. The implemented measures proved effective, reaching a R*_eff_* of 0.67 by 7 April [4]. However, because of the social and economic consequences, the need to reopen parts of society became urgent.

## School closures and transmission of SARS-CoV-2

Evidence for the effect of school closures on disease transmission is mainly based on influenza studies. School closures are most likely to be efficient if the virus has a low reproduction number (R < 2) and if attack rates are higher in children than in adults [5]. However, although estimates vary widely, the R_0_ of severe acute respiratory syndrome coronavirus 2 (SARS-CoV-2) is estimated to be between 2.2 and 3.6 [6-8]. Children are drivers for influenza transmission, but seem to contribute less to the spread of COVID-19 [5,9]. Countries where schools and daycare institutions remained open have not reported outbreaks among children, only sporadic cases [10].

In Norway, as well as globally, the proportion of children with COVID-19 has been low [9,11]. By 11 May, 8,135 COVID-19 cases were reported to the Norwegian Surveillance System for Communicable Diseases (MSIS), of which 72 (0.9%) were aged 0 to 5 years, 162 (2.0%) were aged 6 to 13 years, 341 (4.2%) were aged 14 to 19 years and 7,560 (93.0%) were over 19 years (Figure).

Based on the current evidence and children’s fundamental rights [12], the government announced a gradual reopening of the society, starting with children’s daycares 20 April, primary school grades 1 to 4 on 27 April and higher grades (5 to 13) on 11 May. In order to help schools reopen in a secure manner, the NIPH and the Norwegian Directorate for Education and Training (NDET) were asked to develop specific infection prevention and control (IPC) guidelines. Our guidelines consist of practical IPC advice and assessment of paediatric conditions with risk of severe COVID-19 in terms of school attendance.

## Guidelines for infection prevention in primary schools

The IPC guidelines were developed for primary schools (grade 1–7, children 6–13 years of age) to apply during the COVID-19 epidemic, and were nationally regulated by law [13]. However, local adaptation was encouraged with assistance of local health authorities.

We reviewed the recommendations from the United Nations Children's Fund (UNICEF), the World Health Organization (WHO) and the International Federation of the Red Cross (IFRC) [14], as well as guidelines developed by public health authorities in Canada, Denmark, the United Kingdom and United States available online [15-18]. The guidelines were all useful. However, they did not specifically address how physical distancing could best be implemented in a school setting while still securing children’s need for care and to a certain extent, closer physical contact in the educational setting. Our guidelines also followed the main principles enforcing (i) self-isolation of sick children/staff, (ii) hygiene measures and (iii) physical distancing measures. In addition, schools were required to establish procedures for students or staff who develop symptoms at school. Measures for enforced hand hygiene, respiratory hygiene, cleaning and disinfection will not be further discussed here. For details, see the Supplementary Material. 

We recommended establishing smaller, fixed groups of children and employees, in this setting called ‘cohorts’ as the key physical distancing measure. Reduced contact with others will limit the risk of transmission from presymptomatic and asymptomatic individuals. Establishment of cohorts takes into account that adhering to physical distancing measures is difficult for children and that physical contact is important for children’s development and wellbeing. The cohort strategy ensures physical distancing between cohorts while allowing children’s need for care. Within cohorts, the reduced number of children compared with ordinary classes provides more space and limits the number of contacts. Normally, one cohort is present in the classroom at the time. 

The cohort strategy additionally enables rapid and easy contact tracing, and reduces the need for home quarantine. The identification of contacts between pupils is of high importance for appropriate screening and implementation of preventive measures for affected families and society [19]. With good management, a positive case will only affect the cohort and not the entire school, thereby preventing full school closure.

Cohort size was based on children’s age and the need for care, as well as national regulations for teacher-pupil ratios; up to 15 pupils per teacher in grades 1 to 4 and 20 pupils per teacher in grades 5 to 7. As older pupils can better comply by infection prevention measures, we suggested that groups of older pupils may be somewhat larger. The organisation of cohorts is described in Table 1.

**Table 1:** Organisation of cohorts for physical distancing in primary schools during COVID-19 pandemic, Norway, 2020

Grade (age)	Organisation
**1 to 4 (6–10 years)**	- As a general rule, one staff member should accompany the cohort - The cohorts should minimise changing classrooms - Within a cohort, pupils and staff can socialise and play together - Separate desks 1 m apart recommended - Cohorts should also be maintained in after-school programmes - Cohorts 1 and 2 can work together for practical reasons during the day, preferably outdoors - Staff from cohort 1 can provide relief in cohort 2, and vice versa - Cohorts 3 and 4, and so on, should be organised in a similar way - Cohorts 1 and 2 should generally not mix with cohorts 3 and 4, and so on - Cohorts that are not working together have separate areas or different time points for outdoor activities - Cohorts that are not working together can mind each other and be in the same area for short periods of time (up to 15 min) - Cohorts that are not working together can remain in the same room, provided that a distance of at least 2 m can be maintained between the cohorts over a long period of time - The composition of cohorts can be altered weekly after a weekend
**5 to 7 (11–13 years)**	The recommendations given above apply, in addition to the following: - Teachers can teach in different classes, but cohorts should remain in the same classroom - Cohorts should move between classrooms as little as possible - Pupils and staff within a cohort must strive to stay 1 m apart wherever possible - Consider in-school teaching combined with digital education at home

In addition, we recommended to promote outdoor teaching, and to use larger rooms and facilities when possible. We also recommended that areas and situations with potential for crowding receive special attention regarding the possible need for additional measures to maintain distance. School assemblies, sports games and other gatherings were not advised. Other possibilities for reducing the number of pupils present were staggering the beginning and end of the school day or attendance on different days.

To support school administrators in implementing routines for IPC, we developed a checklist tool for school owners and staff (Table 2, Supplementary Material).

**Table 2:** Checklist for school administrators to ensure infection prevention and control in primary schools during COVID-19 pandemic, Norway, 2020

The school owner’s overarching responsibility
**Train staff regarding infection control measures**
Information for parents/guardians concerning new routines at schools/after-school programmes
Prepare plan for hand washing procedures for pupils and staff
Prepare written procedure for cleaning of premises
Prepare plan for establishment and organisation of cohorts
Establish dialogue with any staff who are in a risk group and children who require special provision
**Hygiene measures**
Ensure sufficient soap and paper towels are available at all handwashing stations and toilets
Training of pupils in handwashing procedures and respiratory hygiene
Put up posters about handwashing procedures and respiratory hygiene
Provide alcohol-based disinfectants where no handwashing facilities are available
Plan hand hygiene measures to be applied outside or on excursions (wet wipes and alcohol-based disinfectants)
**Physical distancing measures**
Consider the use of rooms relative to the number of pupils in the cohorts
Plan for outdoor activities, including staggered times for different cohorts
Divide outdoor areas so that pupils from different cohorts do not mix insofar as is possible
Avoid large gatherings of pupils
Ensure that sufficient stationery and other equipment/materials is available to limit sharing
Provide a separate desk/chair per pupil with a safe distance between pupils
Provide a separate seat for each pupil during meals and activities, with a safe distance between pupils
Ensure distance between pupils at meals and serving food at the table while children are seated
Plan to reduce crowding in changing rooms, toilets and premise entries and exits
If appropriate, apply markings to floors to ensure safe distances are maintained in areas where crowding may occur
Plan for alternating times for breaks to limit the number of pupils who are outside at the same time
Plan for additional adults to be out at break times in order to help pupils maintain a safe distance from each other
Plan for dispersed places where people can assemble before the start of the school day in order to avoid crowding
Plan school transport (school buses, need for additional capacity)
Avoid using public transport for school trips
**Cleaning**
Draw up a cleaning plan, which describes the frequency and methods to be used for the various points; the plan must cover toilets, washbasins and frequently touched objects (door handles, stair banisters, light switches, etc)
Draw up a plan for cleaning toys, tablets, etc.; toys and items that cannot be cleaned must be tidied away
**Recommendations for staff**
Limit physical meetings, arrange video conferencing where appropriate
Maintain social distancing during breaks
Establish procedures for cleaning shared tablets, computers/keyboards
Limit use of public transport

## Recommendations for children and staff at risk for severe COVID-19

Publications on the COVID-19 pandemic report that most children develop mild disease, even those with severe underlying conditions [20-22]. The typical comorbidities associated with severe COVID-19 in adults, particularly diabetes mellitus and hypertension, are associated with increasing age and are not observed in children [23]. The Norwegian Government requested guidelines for school attendance for children with chronic, severe underlying conditions before reopening schools. For this, the NIPH collaborated with the Norwegian Paediatric Association (NPA). A short background document was prepared, and an inquiry conducted between 8 and 13 April to all Paediatric Department Heads at hospitals and NPA-subspecialist committees. Paediatric conditions were evaluated in terms of risk of severe COVID-19 vs depriving children of education and social development. There was a paucity of experience and peer-reviewed publications on this topic from other countries. However, based on available evidence and expert opinion, NIPH and NPA suggested that most children can and should attend school, and that very few conditions justified preventative homeschooling. The NPA published the list of these conditions on their website [24] (Table 3, Supplementary Material).

**Table 3:** List of common paediatric conditions where school attendance is encouraged (left column) and severe conditions where preventative homeschooling can be considered (right column) during COVID-19 pandemic, Norway, 2020

Paediatric conditions where school attendance is encouraged	Paediatric conditions where preventative homeschooling can be considered^a^
- Diabetes mellitus - Non-severe asthma - Allergic conditions - Epilepsy - Cardiac conditions without heart failure - Autoimmune conditions in a stable phase - Solid organ transplant patients in a stable phase - Children with Down syndrome	- First months following solid organ transplantation - First 12 months after stem cell transplantation - Cancer patients during active chemotherapy - Severe cardiac conditions with pulmonary hypertension, heart failure or Fontan circulation - Severe lung diseases and/or reduced lung capacity including need for respiratory support - Severe primary immunodeficiency - Autoimmune disease requiring considerable immunosuppression or in unstable phase - Severe liver failure or renal failure - Other rare conditions may also be considered

^a^ These conditions may require homeschooling in certain periods or on occasion regardless of the COVID-19 pandemic.

School staff with high risk for severe COVID-19 also needed recommendations for when preventive self-isolation was indicated. Knowledge on risk factors was assessed in a rapid literature review by the NIPH [25]. Advanced age (> 65 years) was identified as the main risk factor, especially in combination with comorbidities, with the risk increasing with age. Diabetes mellitus and cardiovascular disease were also considered to possibly represent a risk factor in adults < 65 years. The NIPH recommended that individuals above the age of 65 years may continue preventative self-isolation, while other adults needed to consult their physician to assess individual risk. Employees at risk can still contribute to school education by working from home if possible.

## Discussion

Education is one of the strongest predictors of a population’s health and prosperity, and the impact of long-term school closures has not been evaluated [5]. Children have a right to attend school, which is crucial to their social, physical and psychological wellbeing [12].

The evidence for the effect of school closures on the reduction of COVID-19 disease burden is limited [5], while the negative consequences of school closures include the real risks of deepening social, economic and health inequities [26]. The government therefore decided to reopen schools after 6 weeks of closure. Our guidelines aimed to facilitate the process by providing practical support for schools and information to the public. There was a clear need to evaluate the potential risk for children with severe underlying conditions to ensure safe return to school, and communicate the conclusions to the public. We believe our guidelines may be of value for other countries that plan to reopen schools in the near future.

There was substantial concern about reopening schools among the population, and also among teachers and parents. Based on feedback from teachers’ unions and media reports, the guidelines were perceived as reassuring, providing a manageable framework for safe reopening.

There is an urgent need to evaluate the effect of school closures on disease transmission vs the negative effects on children in the context of the COVID-19 pandemic. This is of paramount importance for possible future surges of COVID-19 as well as for future epidemics. In order to evaluate the effects of school opening on SARS-CoV-2 transmission, pupils and teachers will be prioritised for testing as part of the national surveillance strategy. In addition, a study is planned to examine the transmission of SARS-CoV-2 between children in daycare and primary school settings. This will allow us to better evaluate the effect of implementing IPC when reopening schools.

## Supplementary Data

1682000 Supplementary Material
