# Supplementary material for: Infection prevention guidelines and considerations for paediatric risk groups when reopening primary schools during COVID-19 pandemic, Norway, April 2020
Source: Euro Surveill. 2020 Jun 4;25(22):2000921. doi: 10.2807/1560-7917.ES.2020.25.22.2000921 (PMC7336110; doi:10.2807/1560-7917.ES.2020.25.22.2000921)
Supplement: Supplementary Material [file 20-00921_JOHANSEN_Supplement.pdf]

Supplementary Material

This supplementary material is hosted by Eurosurveillance as supporting information alongside the article [Infection prevention, guidelines and considerations for paediatric risk groups when reopening primary schools during COVID-19 pandemic, Norway, April 2020] on behalf of the authors who remain responsible for the accuracy and appropriateness of the content. The same standards for ethics, copyright, attributions and permissions as for the article apply. Supplements are not edited by Eurosurveillance and the journal is not responsible for the maintenance of any links or email addresses provided therein.

COVID-19 EPIDEMIC:

Guide to infection control for school years 1–7

2nd edition, 7 May 2020

Published by the Norwegian Directorate of Health. Guidelines provided by the Norwegian Institute of Public Health and the Norwegian Directorate of Education and Training

IS-2915: English version guide to infection control for school years 1–7

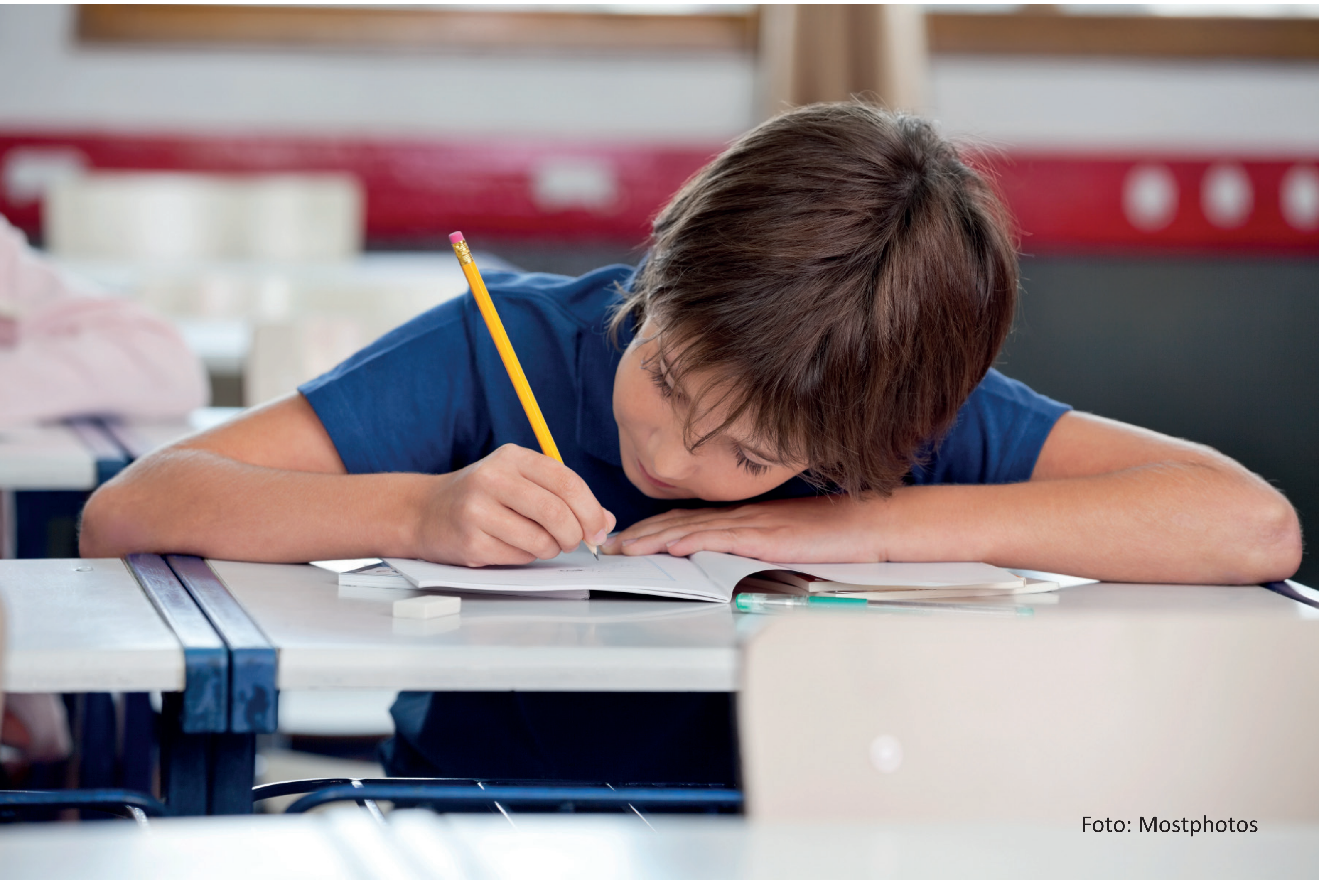

Foto: Mostphotos

The COVID-19 epidemic:
Guide to infection control for school years 1–7

Guidelines provided by
the Norwegian Institute of Public Health and
the Norwegian Directorate of Education and Training
Published by the Norwegian Directorate of Health

2nd edition, 7 May 2020

Contents

1 Introduction ______ **4**

2 Background ______ **5**

2.1 The role of schools in the community 5

2.2 About the virus, the disease and the outbreak 5

2.3 The role of children in the outbreak 6

2.4 The risk of children becoming infected 6

2.5 The risk of children infecting others 6

3 Infection prevention measures ______ **7**

3.1 No sick people should enter schools or attend after-school programmes 7

3.2 When should pupils, parents/guardians and staff attend school, and when can they attend after-school programmes? 8

3.3 When should pupils and staff not attend school/after-school programmes? 8

3.4 When illness occurs at a school/after-school programme 9

3.5 What if someone in the household of a pupil/staff member is ill? 9

3.6 What if someone in the household of a pupil/staff member has been confirmed as having COVID-19? 9

3.7 Practise good hygiene 10

3.7.1 Good hand and respiratory hygiene 10

3.7.2 Good cleanliness 11

3.8 Follow social distancing rules 12

3.9 Infection control measures in certain subjects 17

4 Is there anything which children and staff must pay particular attention to? ______ **18**

4.1 Children with chronic diseases 18

4.2 Adults (parents/guardians /employees) 19

5 Training of staff and information for parents/guardians ______ **20**

5.1 Interaction with parents/guardians and parent meetings 20

6 Visits to schools in spring 2020 for school starters ______ **21**

7 Checklist for infection control at schools and after-school programmes ______ **22**

8 References ______ **24**

1 Introduction

This guide gives advice and guidance to primary schools (years 1-7) which are open during the coronavirus outbreak (COVID-19) in the spring of 2020. The guide is in addition to existing rules and guidelines set out in the "Regulation relating to environmental health in kindergartens and schools, etc." (*Forskrift om miljørettet helsevern i barnehager og skoler m.v.*)[1] [2]. The guide is valid from the date of publication, 20 April 2020. The development of the outbreak and practical considerations may render it necessary for changes to be made at a later date.

It is the school owner that is responsible for ensuring that the school is managed in accordance with applicable regulations[3]. The school owner is responsible for establishing internal control procedures which ensure that the environment in the school promotes health, well-being, good social and environmental conditions and prevents illness and injury[4]. The school owner is therefore responsible for ensuring that the school is managed in a way which addresses the need for infection control. However, it is important to stress the need for everyone to contribute to the appropriate management of schools during the COVID-19 outbreak. Implementing appropriate infection control measures in a school which is open involves collaboration between all those involved in running the school. It requires good cooperation between the staff at the school and the school health service, as well as the cleaners. A good dialogue between the school and households will also be vital.

The guide is based around the applicable framework conditions, regulations and funding, including regulations pursuant to the Corona Act (*koronaloven*).

This guide has been prepared by the Norwegian Institute of Public Health and the Norwegian Directorate of Education and Training. The guide was prepared on behalf of the Ministry of Education and Research and the Ministry of Health and Care Services, via the Norwegian Directorate of Health.

Updated 2nd edition published on 7 May 2020. Changes in this edition are as follows:

- Further explanation of cohort organisation
- Advice concerning the cleaning of outdoor toys
- Clarification of certain advice
- Advice for recipients of school starters

[1] https://lovdata.no/dokument/SF/forskrift/1995-12-01-928
[2] https://www.helsedirektoratet.no/veiledere/miljo-og-helse-i-barnehagen
[3] see Section 13-10 of the Education Act (*opplæringsloven*) and Section 5-2 first paragraph of the Independent School Act (*friskoleloven*).
[4] https://lovdata.no/dokument/SF/forskrift/1995-12-01-928

2 Background

In spring 2020, a global outbreak of the coronavirus SARS-CoV-2 led to the implementation of a series of strict measures to limit the spread of the virus through infection. On Thursday 12 March 2020, it was decided that all kindergartens and schools should be closed from 6pm on the same day until Thursday 26 March 2020. The measures were reviewed on an ongoing basis, and on Tuesday 24 March, the government decided that all kindergartens and schools should remain closed until 13 April.

On 7 April 2020, the government approved the gradual reopening of kindergartens and schools. The Norwegian Institute of Public Health (NIPH) and the Norwegian Directorate of Education and Training (NDET) are aware that many staff at kindergartens and schools, as well as parents and guardians, are unsure how kindergartens and schools can be reopened safely and how they can best protect against infection. This guide is intended to provide an insight into and examples of how school and after-school programmes can be organised, while at the same time ensuring that infection control measures are implemented. This guide applies to all primary schools, regardless of the basis on which the school is run[5]. Regarding infection control measures relating to pupils with special needs, we also refer to the measures described in the guide for kindergartens where applicable.

2.1 The role of schools in the community

Schools play a crucial role in children's learning, care and development. Children and young people have both a right[6] and an obligation to attend primary and secondary education when they are likely to be staying or living in Norway for more than three months. The UN Convention on the Rights of the Child, the Norwegian Constitution[7] and the regulations within the field of education all recognise the importance of schools as an arena for children's learning, development and well-being. Schools are also vital to the structure of society, as they enable parents and guardians to go to work. School staff therefore also play a pivotal role in looking after pupils in a pandemic situation. The outbreak could last for a long time, depending on the infection control measures which are implemented within the population at large. It is therefore vital that children are able to attend school during the outbreak period, and that infection control measures are implemented covering both pupils and staff alike. To limit the spread of infection during the controlled reopening of schools, there are a number of measures that schools can implement.

2.2 About the virus, the disease and the outbreak

The disease COVID-19 is caused by the SARS-CoV-2 virus, commonly known as novel coronavirus. The virus can cause respiratory infection of varying severity. It is mainly transmitted via droplet and contact infection, where the virus in the respiratory tract is transmitted from the respiratory tract of a sick person to another person. Infection can therefore occur both if you cough or sneeze at another person, or if you get respiratory secretions on objects which other people then touch. Some people may have COVID-19 without noticing any symptoms. It is currently unclear what role they play in the spreading of infection. At the present time, it is believed that people can be infected for 1-2 days before they develop symptoms, even though people are most contagious while they

[5] See Section 2-1 of the Independent School Act.
[6] Right to public primary education; see Section 2-1 of the Education Act.
[7] Section 109 of the Norwegian Constitution

are experiencing symptoms, particularly during the first few days. The virus can be killed by soap and water, or disinfection using alcohol, sunlight or temperatures above 60°C.

2.3 The role of children in the outbreak

There are a number of knowledge summaries concerning the effects of school closures in order to limit the spread of infection, but these are based on research relating to influenza epidemics. The results indicate that although school closures may limit the spread of infection, the magnitude of the impact is difficult to estimate. The transferral value of these results to the COVID-19 pandemic is uncertain, particularly because children are playing a less important role in the spread of infection than they do in influenza epidemics. It is currently believed that the overall negative impacts of closing kindergartens and schools far outweigh the possible benefits as regards limiting the spread of infection[8].

2.4 The risk of children becoming infected

Compared with adults, few children have been identified as having been infected in either Norway or elsewhere in the world. As of 6 May 2020, a total of 7,953 laboratory-confirmed cases had been recorded in Norway, of which 130 were aged 0 to 9 years (1.6%), and 418 were aged 10 to 19 years (5.3%) (www.fhi.no). In Iceland, where no schools have been closed, a large sample of the population was tested for COVID-19 in March 2020, and amongst 848 children under the age of 10 without any symptoms, no one had any demonstrable infection. So far, this suggests that children become infected to a lesser extent than adults[9].

2.5 The risk of children infecting others

Our current knowledge indicates that children do not play as important a role in the spreading of COVID-19 amongst the general population as adults. Children can still become infected and experience symptoms. It is primarily children and adults with symptoms who are contagious, and the contagiousness is greatest when the symptoms are developing. As children have less symptoms than adults, their contagiousness will also be lower. Before schools and kindergartens were closed in Norway, only sporadic cases of infection were reported amongst older children. Sweden, Iceland and Finland, whose schools are open, are recording few cases amongst children and have so far not recorded any outbreaks in schools or kindergartens. It is reassuring to know that there have so far been few or no known outbreaks in schools or kindergartens during the epidemic, but it must be remembered that many countries closed their schools at an early stage. For more information, see www.fhi.no.

[8] https://www.fhi.no/publ/2020/barns-rolle-i-spredning-av-sars-cov-2-covid-19-1.-oppdatering/
[9] https://www.nejm.org/doi/full/10.1056/NEJMoa2006100?query=featured_home

3 Infection prevention measures

Many measures are being implemented throughout society which are collectively limiting the spread of infection. It is important to use measures which are adapted to different situations. It is therefore not always possible to use the same measures in schools, businesses and the population at large. The measures must be adapted in order to focus on what is most important in each individual situation. Here, we describe measures to limit the spread of infection amongst pupils in years 1-7 and staff.

No one can be blamed if an infection occurs in a school. For as long as the virus is circulating in society, disease can arise because it is not possible to prevent every single new case. This means that infection may enter a school, but the measures described here will limit the spread of infection amongst children and staff in schools.

The aim of the advice is to limit the spread of COVID-19. In order of priority, the three cornerstones for limiting the spread of infection are:

1. No sick people should enter schools
2. Practise good hygiene
3. Follow social distancing rules

The most important infection control measure is for sick people to stay at home. Good cough etiquette and social distancing are essential for limiting droplet infection, while hand hygiene, particularly avoiding touching your face with dirty hands, is important to prevent indirect contact infection. Maintaining a greater physical distance between people reduces the possibility of infection, even before symptoms of disease develop.

Even if measures are implemented properly, cases of COVID-19 can still occur. Nevertheless, implementing the infection control measures listed here will limit the spread of infection. The measures described below explain the infection control measures that must be in place in order to successfully reduce infection risk to a minimum.

The municipal health service possesses infection control expertise and can be consulted regarding questions about organisation and facilitation at local level.

3.1 No sick people should enter schools or attend after-school programmes

The symptoms of COVID-19, particularly in children, can be mild and difficult to distinguish from other respiratory infections. COVID-19 is mainly transmitted via droplet and contact infection. It is primarily people with symptoms who are contagious, and they are most contagious while their symptoms are developing.

The most frequently described symptoms of COVID-19 during the early stages of infection are a sore throat, cold and mild cough, as well as general malaise, headache and muscle pain. Abdominal pain may also occur.

Approximately 8 out of 10 adult adults only experience mild symptoms. Amongst children, this proportion is probably even higher. In some people with COVID-19, symptoms can develop further within a week to a cough, fever and shortness of breath, and a very small number of people may experience severe symptoms and require treatment in hospital. Severe COVID-19 disease is very rare in children.

The municipal health service is responsible for monitoring cases of confirmed COVID-19 (infection tracking) and must notify the school if children or staff at a school are confirmed

as having COVID-19. The municipal health service must decide on the measures that are necessary, e.g. defining who has come into close contact with the sick person and must therefore go into quarantine, and whether any information should be given to anyone else. If further measures are necessary, the municipal health service is responsible for issuing orders to the school.

Further information is given below regarding when pupils, parents/guardians and staff may or may not be at school and be involved with after-school programmes.

3.2 When should pupils, parents/guardians and staff attend school, and when can they attend after-school programmes?

Pupils and staff should attend school/after-school programmes:

- When they are not experiencing any symptoms of the disease.
- If they have had a respiratory infection which has now passed, children and staff may return to school after being symptom-free for 24 hours.
- If anyone in their household is experiencing the symptoms of respiratory infection but has not been confirmed as having COVID-19, pupils and staff must attend school/after-school programmes as normal. However, these people should go home from school/after-school programme if they experience any symptoms (see below).
- Pupils and staff with typical symptoms of pollen allergy (known pollen allergy, runny nose with clear nose secretion, runny/itchy eyes) may attend school/after-school programmes.
- Many younger children often have a runny nose without any other symptoms of respiratory infection, particularly after they have been outside. These children may attend school/after-school programmes if they do not have a fever and are otherwise healthy.

Parents/guardians who are healthy and do not have symptoms of respiratory infection can take and collect their children to/from school/after-school programme as normal.

3.3 When should pupils and staff not attend school/after-school programmes?

It is important that parents/guardians are notified and understand the reasons why children who are experiencing symptoms should not attend school/after-school programmes.

Pupils and staff with respiratory symptoms:

- Pupils and staff should not attend school/after-school programmes even if they are only experiencing mild respiratory symptoms and general malaise. They should stay at home until they have been symptom-free for 24 hours.

Students or staff with confirmed COVID-19:

- Must be in isolation. The health service is responsible for deciding who should self-isolate and for how long based on advice issued by the NIPH (see www.fhi.no). Guidelines to the isolation regulations are issued by the Norwegian Directorate of Health.

Pupils and staff who are a close contact of or a household member with a person with confirmed COVID-19:

- Must be quarantined. The health service is responsible for deciding who should be quarantined and for how long based on advice issued by the NIPH (see www.fhi.no). Guidelines to the quarantine regulations are issued by the Norwegian Directorate of Health.

Parents/guardians who are experiencing respiratory symptoms or are in quarantine or isolation:

- Must not attend school/after-school programmes, and must find other solutions for taking and collecting their children. Parents/guardians who have had COVID-19, but have now ended their isolation in accordance with the health service's recommendations, can attend school as normal.

3.4 When illness occurs at a school/after-school programme

It is important that pupils who experience symptoms of respiratory infection go home (after their parents/guardians have been notified) or are collected as soon as possible. Sick pupils should not travel on public transport. Pupils who have to be collected must wait in a separate room together with a member of staff or outside where there are no other children around. Where possible, the member of staff should keep at least two metres away, but a balance must be struck in relation to the age of the pupil and their care needs. If it is not possible to maintain a distance of two metres and the pupil is comfortable doing so, the pupil should put on a mask to reduce the spreading of infection to others. If the pupil does not wish to wear a mask, the adult should put on mask, unless a distance of two metres can be maintained. If a visit to the toilet is necessary, the toilet should then not be used by anyone else until it has been cleaned. The person looking after the pupil must wash their hands after they have spent time with the pupil. The room, toilet and other areas where the pupil has been must be cleaned afterwards using ordinary detergents.

Staff members who fall ill while at school/after-school programme must go home as soon as possible. The pupils for whom the sick member staff is responsible must be looked after by other members of staff/deputies or sent home. Sick staff members should not travel on public transport. Sick staff members should wear a mask to reduce the spreading of infection if they are unable to maintain a distance of two metres away from other people.

3.5 What if someone in the household of a pupil/staff member is ill?

If someone in their household is experiencing the symptoms of respiratory infection but has not been confirmed as having COVID-19, the pupil/staff member must attend school as normal. The pupil may also attend after-school programmes. However, the pupil/staff member should go home from school/after-school programme if they experience any symptoms referred to in the section above. Other hygiene measures outlined in the following chapters will reduce the risk of spread of infection.

3.6 What if someone in the household of a pupil/staff member has been confirmed as having COVID-19?

If another household member has been confirmed as having COVID-19, all those who have come into close contact with them must go into quarantine in accordance with the health

authorities' recommendations. No one who is in quarantine themselves must attend school or after-school programmes.

Flowchart for dealing with sick children at primary schools and after-school programmes

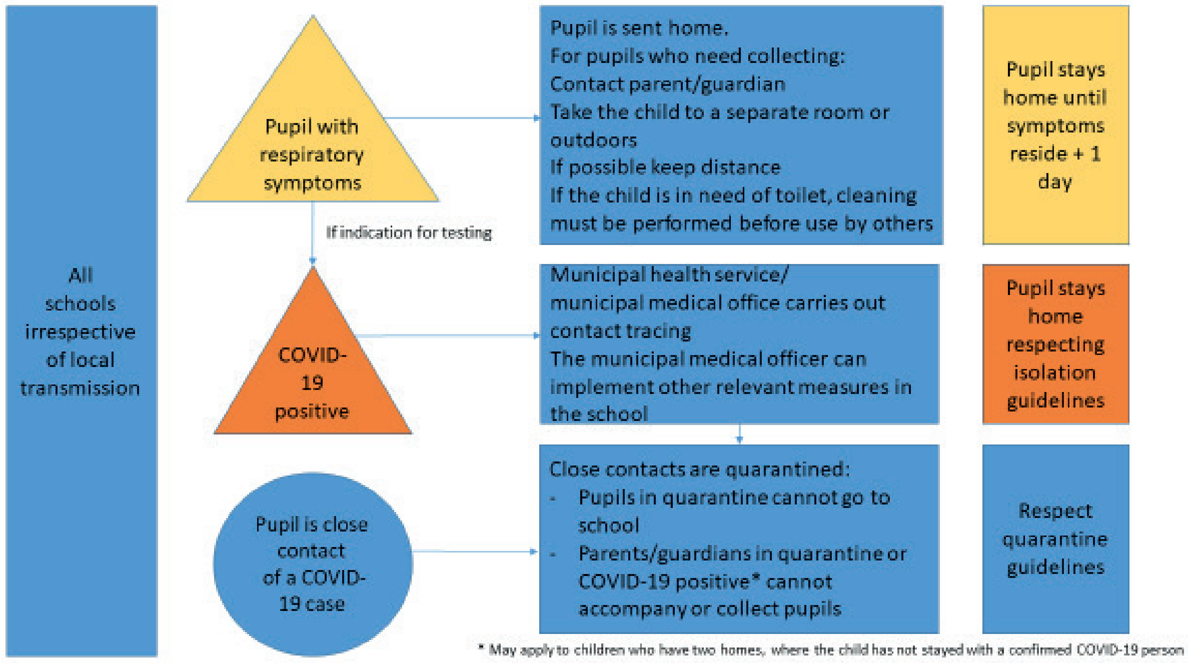

3.7 Practise good hygiene

3.7.1 Good hand and respiratory hygiene

Good hand and cough respiratory hygiene (cough etiquette) reduces infection in connection with all respiratory infections, including COVID-19 infection. These measures should reduce infection via objects and hands, and reduce infection via coughing. Ensuring that your hands are clean is the most important measure that can be taken to prevent such indirect infection.

Hygiene measures must be carried out frequently by everyone, regardless of what they know about their infection status or that of other people around them. It is important that all pupils and staff are familiar with the measures below.

No special personal protective equipment is required, other than to have non-medical face masks available to pupils or staff who become ill where it is not possible to maintain a distance of two metres.

Regarding pupils with special needs, e.g. where nappy changes are necessary, see the guide for kindergartens.

Hand washing with lukewarm water and liquid soap is an effective way of preventing infection. Dirt, bacteria and viruses detach from the skin during washing and rinse away with the water. Pupils and staff must wash their hands frequently and thoroughly for at least 20 seconds. Hands must then be dried using disposable paper towels. Both staff and pupils must be given adequate training in hand washing. This can be reinforced by giving verbal reminders and by putting up posters with instructions on good hand washing practices.

As regards the youngest pupils, procedures can be created which the children find enjoyable to follow. As a general rule, hand washing should take place with the adult present, particularly in the case of the youngest pupils.

Pupils and staff must wash their hands:

- Before leaving home and when they get home
- When arriving at school/after-school programme
- After coughing/sneezing
- After going to the toilet
- Before and after meals
- After coming in from outdoor activities/breaks
- When their hands are visibly dirty

Alternatives to hand washing:

- The virus is sensitive to alcohol, and using alcohol-based disinfectants (hand sanitizer) is an option unless hand washing facilities are available.
- Hand disinfectant must be provided in places where it is not possible to wash your hands (e.g. in changing rooms).
- Alcohol-based disinfectants are ineffective on wet or visibly dirty hands, when hand washing should be carried out.
- Alcohol-based disinfectants are suitable for use outside and when away from the home, after dirty hands have first been cleaned using wet wipes.

Cough etiquette and respiratory hygiene:

- It is recommended to paper towels be provided in a readily accessible place for use by pupils/staff. If paper towels are not available, it is recommended that pupils/staff cough or sneeze into their elbow when necessary.
- Avoid touching the face or eyes. This can be difficult for young children, but older pupils and staff should be encouraged to follow this advice.

3.7.2 Good cleanliness

Novel coronavirus (SARS-CoV-2) is easily removed by manual cleaning using water and ordinary detergents. The virus can survive on surfaces for anything between a few hours and a few days, depending on the type of surface, temperature, sunlight and other factors. Thorough and frequent cleaning is therefore important in order to prevent infection.

Exposed areas (see below) should undergo extra cleaning. Review procedures and local cleaning plans, and make any necessary adjustments (organisation, responsibilities and resource requirements).

It is not necessary to use additional protective equipment. Wash hands after cleaning has been carried out, even if gloves have been worn. Loose carpets should be removed to facilitate cleaning.

It is not necessary to use disinfectants routinely at school. However, if disinfectants are used, visible dirt must first be wiped off using a cloth or paper towel; otherwise, the disinfectant will not work. Alcohol- and chlorine-based disinfectants may be used.

It is not necessary to clean outdoor play equipment or toys which are used outdoors. The most important consideration is to wash your hands before and after playing outdoors, and before eating. The virus is also inactivated by sunlight.

Extra cleaning is important in exposed areas:

- Toilets and washbasins must be cleaned at least daily. Surfaces such as toilets seat and washbasin taps must be wiped down 2-4 times a day.
- Disposable paper towels and soap should be provided and refuse bins must be emptied regularly.
- Dining tables must be cleaned using water and soap after use.
- Door handles, stair banisters, chairs, other table surfaces and other items that are often touched must be cleaned frequently, at least daily.
- Pupils must clean their own desk daily.
- Toys, tablets, computers/keyboards which are shared must also be cleaned, at least daily.
- Reduce the number of toys in order to make cleaning more feasible.
- Toys and equipment which are only used by one cohort do not need to be cleaned daily.
- As an alternative to daily cleaning, toys and equipment can also be tidied away ("quarantined") for three days before they are reused.
- Play costumes and other loose fabrics must be washed according to the relevant instructions. If this is not possible, they must be tidied away.
- Tableware and cutlery must be washed in a dishwasher in accordance with the applicable procedures.

3.8 Follow social distancing rules

Social distancing will reduce the risk of infection from people who do not know they are contagious. To achieve this, you can increase the distance between people and/or reduce the number of people who are close together. Major events must be organised in accordance with the applicable guidelines from the Directorate of Health. It is important to stress that staff will continue to attend to their pupils' needs regarding contact and care.

As regards the youngest pupils/pupils with special needs, it is difficult to control the distance they maintain from other people. In this case, the most important measure will be to limit the number of close contacts of each pupil and staff member, for example by having smaller groups and designated staff members for each group/class. Older pupils find it easier to follow advice concerning distances and other infection control measures, and their groups can therefore be somewhat larger.

The aim of establishing fixed groups is to ensure that everyone can comply with the social distancing requirements within their groups, and to limit contact with other groups. This will limit the spread of infection both within a group and between groups. It will also make the task of infection tracking easier and prevent the entire school from having to close when a pupil or staff member is confirmed as being infected.

A fixed group of pupils and staff is known as a "cohort" and, in school/after-school programmes, such cohorts can be a good starting point for limiting and tracing any infection. Cohorts could for example be based around the school's existing class structure, the number of pupils in the year and the standard requirement regarding teacher-pupil

ratios (15 pupils per teacher in years 1 to 4, and 20 pupils per teacher in years 5 to 7)[10]. Cohorts must have as little interaction with other cohorts as possible. Within a cohort, pupils and staff can socialise and play together. However, the oldest pupils in particular must be encouraged to maintain a distance of at least one metre and avoid unnecessary close contact. The youngest pupils will not find this as easy to follow.

The basis for cohort sizes should be the ratio between teachers and pupils, but larger cohorts may be established. For such cases, some practical considerations are given below which must be taken into account when determining cohort sizes:

- Cohort sizes can be adapted to local circumstances, the size of the year, and the individual school's circumstances.
- For practical implementation, it is possible to increase the size of a cohort size by a few extra children. Schools must themselves consider whether it is prudent to increase the size of cohorts based on the age of the pupils, the level of activity, the need for adult supervision and the ability to maintain a safe distance between pupils.
- The layout of the premises will influence the number of pupils and staff who can be present in the same room at the same time, so that it is possible to maintain a safe distance between pupils and staff.
- There must be one adult per cohort.
- Cohorts should also be maintained in after-schools programmes wherever possible.

Staff members should go to the classroom, so that the cohort does not need to change rooms. Cohorts should also change classrooms as little as possible.

Two cohorts can work together if necessary for practical purposes during the day and so that staff can have a break. Such cooperation should preferably take place outdoors (joint outdoor teaching, play, excursions and sports). It is easier to maintain a safe distance during outdoor activities, and the virus is also inactivated by sunlight. If indoor cooperation is necessary (joint teaching), sufficient rooms/facilities must be available to ensure that at least one metre can be maintained between pupils/desks. If the number of pupils in a cohort is increased, cohorts should not generally cooperate indoors because it leads to more contact between pupils.

<u>The overriding goal is for pupils and staff to have a limited number of contacts.</u>

In order to avoid overly close contact between cohorts, provision can be made to enable the greater use of outdoor activities. Several cohorts can be outdoors at the same time. Cohorts which are not working together but are outdoors at the same time should preferably use separate outdoor areas. Cohorts which are not working together can take it in turns to use outdoor areas, and the areas need not be cleaned in connection with each changeover. In the event of a shortage of physical space, consideration can be given to scheduling alternating days or staggered attendance times or using alternative premises[11]. When necessary, the composition of cohorts can be altered, but no more than once a week and after a weekend (at least two days without any contact between children and staff in a cohort). This will ensure that close contact is minimised if infection is demonstrated in a child or staff member.

[10] See the Regulation pursuant to Section 14A-1 of the Education Act.
[11] https://lovdata.no/dokument/LTI/forskrift/2020-03-27-470

Pupils should ideally not socialise with many other children in their spare time.

Table: Examples of the organisation of cohorts (a cohort is a group of children and adults as defined in the text above).

Year	Example* of organisation per week
**Years 1 - 4**	• As a general rule, one member of staff should accompany their cohort<br>• Cohorts should move between classrooms as little as possible<br>• Cohorts 1 and 2 can work together for practical reasons during the day, preferably outdoors (see the text)<br>• Staff from cohort 1 can provide relief in cohort 2, and vice versa<br>• Cohorts (who are not working together) should use separate outdoors play areas<br>• Cohorts should also be maintained in after-school programmes<br>• Cohorts 3 and 4, and so on, should be organised in a similar way.<br>• Cohorts 1 and 2 should generally not mix with cohorts 3 and 4, and so on.<br>• Cohorts which are not working together can mind each other and be in the same area for short periods of time (up to 15 minutes)<br>• Cohorts which are not working together can remain in the same room, provided that a distance of at least two metres can be maintained between the cohorts over a long period of time<br>• The composition of cohorts can be altered weekly after a weekend
**Years 5 - 7**	The recommendations given above apply, in addition to the following:<br>• Staff members should go to the classroom, so that the cohort does not have to move from one room to another<br>• Cohorts should move between classrooms as little as possible<br>• Pupils and staff within a cohort must strive to remain at least one metre apart wherever possible

*Supplementary information in the text before table.

Some suggestions are given here as to how contact -reducing measures can be implemented amongst pupils. Each school must find solutions that can be adapted locally to their own particular circumstances. Specific advice for contact-reducing measures for staff will be given at the end.

Physical contact:

- The need for closeness and care must be addressed. Employees must wash their hands after close contact and comforting.
- Shaking hands, hugging and unnecessary physical contact must be avoided wherever possible.

Density in groups:

- Establish cohorts of children (see above).
- The composition of cohorts can be altered weekly after a weekend.
- Use larger premises wherever possible.
- Where possible, staff members should go to the classroom, so that pupils do not need to change rooms.
- All pupils should have their own desk in the classroom to maintain a safe distance between children. Where children do not have their own desk, workplaces should be provided for them which enable them to maintain a safe distance (one metre) away from other children.

- Ensure that all children have their own eating and activity space during after-school programmes, which ensures that children maintain a safe distance from each other.
- Avoid congestion on the way in and out of classrooms and in changing rooms and toilets.
- Alternatively, apply markings to the floor to ensure that a safe distance is maintained between pupils in group rooms/classrooms, changing rooms and other areas that can become congested.
- Make greater use of outdoor time and outdoor schooling. It is recommended that as many activities as possible take place outdoors, particularly in the case of after-school programmes.
- To avoid having several cohorts together, outdoor areas can be divided into zones.
- Locally, it may be appropriate to consider a school and the associated after-school programme in context, in order to facilitate greater flexibility in terms of the timing of lessons and after-school programmes, thereby reducing the group size of children.

At the start and end of the day:

- Agree dispersed places where pupils can assemble before the start of the school day in order to limit the number of pupils gathered together in one place and to avoid congestion on the way in and out of classrooms.
- Parents/guardians must not accompany their children onto the school premises or into changing rooms unless absolutely necessary.

Play and breaks:

- Schedule breaks at different times to limit the number of pupils who are out at the same time. For example, schedule breaks for one or two year groups at a time. This must be assessed based on the number of pupils in each year.
- Arrange for additional adults to be out at break times in order to help pupils maintain a safe distance from each other.

Limit the sharing of food and items:

- Pupils must not share food and drink.
- Food can often be prepared at school/after-school programme, but pupils should not generally be involved.
- Food should be served in portions.
- Pupils should sit at their own desk when eating, or in fixed places with a safe distance between them.
- Children should eat in their cohorts. In the case of shared canteens, cohorts should eat at different times. Tables and chairs must be cleaned after each group.
- Limit the sharing of stationery, tablets and other equipment.
- Learning materials, toys, etc. should not be shared between cohorts until they have been cleaned.
- Textbooks can be taken between home and school, but not shared between pupils.
- Toys should not be brought from home.
- Class teddy bears must be placed in quarantine during the COVID-19 outbreak.

Transport and school transport:

- The use of public transport to and from the school should be limited as much as possible.
- School trips that involve close gatherings of pupils in large groups or on public transport should be avoided.
- Regarding the use of school transport, see the guidance regarding infection control on public transport[12]. Pupils from the same household can sit together. Pupils must remain one metre apart while waiting for the bus or other mode of transport.
- Pupils must wash their hands when they arrive at school and when they return home.

Specifically for staff:

- Maintain a safe distance (at least one metre) from colleagues during meetings and breaks.
- Where different teachers are required, teachers can switch between cohorts, while the cohorts remain in fixed classrooms wherever possible
- Avoid handshakes and hugging.
- Avoid the wearing of rings and hand jewellery at the workplace.
- Use video conferencing instead of physical meetings with colleagues wherever possible.
- Shared tablets, computers/keyboards must be cleaned after use.
- Pay particular attention to hygiene around kitchens/canteens.
- Limit the use of public transport during rush hour to and from the workplace wherever possible.
- Permanent or temporary staff who work at a number of schools must undergo training concerning the applicable local procedures that apply at each school. Temporary staff should avoid working in different schools on the same day.
- In the event of staff absence, temporary cover staff may be required or staff may need to be reassigned between cohorts. This can be done when necessary, but reassignments should be limited as much as possible.
- Special services (Educational and Psychological Counselling Service, speech therapists and others) must follow basic infection control procedures in accordance with the applicable recommendations (good hand and respiratory hygiene, do not go to work when ill, etc.), but can otherwise perform their work in the normal way.

Specifically for the school health service:

- Public health nurses play an important role as advisers regarding infection control at schools. Public health nurses are also important collaboration partners for pupils and with regard to the provision of health-related and psychosocial support in accordance with national guidelines for health centres and the school health service[13].
- Teaching, discussions, surveys, monitoring and vaccination of pupils can be carried out according to the same guidelines as for other health and care services. See www.fhi.no

---

[12] https://www.fhi.no/nettpub/coronavirus/rad-og-informasjon-til-andre-sektorer-og-yrkesgrupper/kollektivtransport/

[13] https://www.helsedirektoratet.no/retningslinjer/helsestasjons-og-skolehelsetjenesten

3.9 Infection control measures in certain subjects

Some advice is given below regarding specific subjects where additional measures are required, in addition to the recommendations that are given above.

Use of changing rooms and the teaching of physical education (PE):

- If appropriate, changing prior to PE lessons may take place in different classrooms in order to provide more space.
- It is recommended that showers after physical exercise be deferred until the pupils have returned home.
- If showers are necessary, the pupils must remain in their cohorts in order to limit the number of pupils in the changing room/shower at the same time.
- The use of outdoor gyms is recommended.
- Avoid activities which involve close contact between pupils, such as some team sports, couples dancing, martial arts, or similar.
- If at a later stage in the outbreak, swimming lessons become relevant: The chlorine in swimming pool water inactivates both coronaviruses and other viruses. Teaching must be organised so as to avoid close contact between pupils in changing rooms and the pool.

Music

- Avoid the sharing of wind instruments between several pupils.
- In connection with the use of handheld instruments and keys, surfaces that are touched must be wiped off after use.

Food and health

- Wherever possible, teaching should be carried out in alternative ways (e.g. through home assignments).
- The virus is not transmitted via food provided good hand hygiene and normal kitchen hygiene are practised.
- Avoid foods which are prepared without heat treatment (e.g. salads, etc.).

Arts and crafts

- Limit the sharing of equipment. Clean equipment after use wherever possible.

School libraries

- Books may be loaned provided that hand washing is practised before arrival at the school library.
- Return of books after illness: the virus can survive on paper/cardboard for 24 hours. Under all circumstances, pupils with symptoms of respiratory infection must stay at home until they have been symptom-free for 24 hours, or longer if they are in quarantine or isolation. Books can therefore be returned when the child themselves is able to return to school.

4 Is there anything which children and staff must pay particular attention to?

4.1 Children with chronic diseases

Children and adolescents have so far not been shown to be at risk of developing severe symptoms of COVID-19. However, the knowledge base is limited and, to reduce the risk of infection, some children may therefore be advised not to attend school as a precautionary measure.

In consultation with the NIPH, the Norwegian Paediatric Association (NPA) has prepared an overview of various diagnoses in children and adolescents and any special considerations which should be taken into account. The NPA believes that the vast majority of children with chronic diseases can and should attend kindergarten or school.

Children and adolescents with a chronic disease/condition who can **attend school** in the normal way, include:

- Children and adolescents with diabetes
- Children and adolescents with well-controlled asthma
- Children and adolescents with allergies
- Children and adolescents with epilepsy
- Children and adolescents with Down syndrome
- Children and adolescents with heart defects without heart failure
- Children and adolescents with an autoimmune disease who use immunosuppressive therapy and have a stable condition
- Former premature children without significant lung disease

Children and adolescents with one or more chronic diseases, where consideration can be given on an individual basis to whether or not there are grounds for the child/adolescent to be kept at home and receive facilitated teaching from school, are listed below (1-8). The school and parents/guardians should discuss whether it is possible to arrange for these pupils to attend school in the normal way. If special provision for regular teaching is not possible, efforts may be made to facilitate participation at certain times during the school day, e.g. in small groups (2-3 pupils) or during outdoor activities. When assessing how the teaching and special provision can be carried out, considerable emphasis must be placed on what would be in the best interests of the pupil[14]. For example, it will be crucial that the benefits of receiving facilitated education at home outweigh the disadvantages to these children as a result of losing out on important social and academic development:

1. Children and adolescents who have undergone an organ transplant (early period following transplant)
2. Children and adolescents who have undergone a bone marrow transplant during the past 12 months
3. Children and adolescents undergoing active cancer treatment
4. Children and adolescents with severe heart disease
5. Children and adolescents with severe lung disease and/or significantly reduced lung capacity

[14] See Article 3 of the Convention on the Rights of the Child

6. Children and adolescents diagnosed with a severe congenital disease of the immune system in an unstable phase which makes them particularly vulnerable to serious respiratory infections
7. Children and adolescents with an active chronic disease who are taking immunosuppressive medication
8. This may also apply to certain other children with serious diagnoses not referred to here. In such cases, the need for facilitated teaching must be clarified with the attending paediatrician.

The diagnoses included in groups 1 - 8 are specified in more detail on the website of the NPA: https://www.legeforeningen.no/foreningsledd/fagmed/norsk–barnelegeforening/veileders/informasjon-veileders-og-procedures-for-barn-og-covid-19/. The list has also been communicated to the Norwegian Association of General Practioners (*Norsk forening for allmennmedisin*).

As a general rule, there is no basis for siblings of these children/adolescents to be kept at home. An important exception is siblings who are scheduled to become a bone marrow donor for a household member (final 28 days prior to donation).

4.2 Adults (parents/guardians /employees)

Based on information available about the outbreak so far from China, Italy, the United Kingdom, the United States and Norway, older people (over 65 years of age) are at greater risk of serious illness with the new coronavirus, particularly if they already have an underlying chronic disease. In addition, adults, especially those over the age of 50, with a chronic disease such as cardiovascular disease (including high blood pressure) and diabetes may be at a slightly higher risk of developing severe symptoms as a result of COVID-19. Staff belonging to groups at greater risk of severe COVID-19 disease should be assessed individually in relation to the possibility of adapted work. Staff being considered for such work must have a medical certificate.

For updated information on those who may be at greater risk of COVID-19, see the NIPH´s website: https://www.fhi.no/nettpub/coronavirus/fakta/risikogrupper/?term=&h=1

Pregnant women have not been shown to be at greater risk of developing severe symptoms of COVID-19 and there is also no evidence to suggest that infection may cause a risk of damage to the foetus. The NIPH recommends that pregnant women who have a chronic illness or pregnancy complications should discuss with their GP whether there is any reason to take additional care or need for facilitation at the workplace.

Parents/guardians who are at risk can contact their GP to assess the need for facilitating school provision/home education in order to limit the risk of infection in the home. For pupils with parents/guardians (others in the household) who are at risk, a dialogue should be established between the school and the parents/guardians concerning whether it is possible to facilitate the teaching so that these pupils can still attend school in the normal way. In such cases, consideration for the child's best[15] interests should also be afforded great importance. If special provision for regular teaching is not possible, efforts may be made to facilitate participation at certain times during the school day, e.g. in small groups (2-3 pupils) or during outdoor activities.

[15] See Article 3 of the Convention on the Rights of the Child

5 Training of staff and information for parents/guardians

Staff must be trained to carry out the necessary infection control measures described in this guide. The guide will be supplemented with additional information which can be used in the training of staff.

Parents and guardians must be confident that it is prudent for their children to return to their school/after-school programme. It is therefore important that the school has a good dialogue with parents and guardians. It is also important that parents and guardians actively contribute to implementing the infection control measures described in this guide. Specific information is being prepared for this group.

Information on this can be found at http://www.udir.no/kvalitet-og-kompetanse/sikkerhet-og-beredskap/informasjon-om-koronaviruset/smittevernveileder/

5.1 Interaction with parents/guardians and parent meetings

Wherever possible, no parent/guardian meetings and other large gatherings should be arranged at this stage of the COVID-19 outbreak. It is recommended that digital meetings take place instead.

Parents' evenings should be replaced by digital meetings. Where this is not possible, parents' evenings can be conducted with the following measures:

- Different families should not wait in the same room
- Wash hands before and after the meeting
- Maintain at least one metre distance between staff and pupils/guardians
- Any interpreters or similar that are required must also maintain a safe distance away from other people

6 Visits to schools in spring 2020 for school starters

School starters come from different kindergartens. This leads to the mixing of children and parents/guardians and requires special considerations. The same key principles of infection control as described above must be applied (no sick persons present, good hygiene, and reduced contact between individuals).

Organisation:

- Depending on the number of school starters each school has, no more than 50 school starters including parents/guardians should be present at the same time.
- If sufficient space is available, two groups of 50 people may be present at the same time, but in different places.
- Children should be divided into groups of up to 15 children in each.
- The children must not have close physical contact with godparents in higher years, and must maintain a distance of at least one metre.
- Where possible, children from the same kindergarten should attend at the same time. Consideration can be given to whether the children can attend school in their cohorts together with kindergarten staff.
- The children should have their own desk in the classroom and be at least one metre away from everyone else.
- If parents/guardians must accompany children, they must also be able to keep at least one metre away from other children and staff. It is recommended that only one parent/guardian accompanies each child.
- Meetings with parents/guardians should primarily take place digitally. If this is not possible or practicable, the information should be provided in premises which allow at least one metre to be maintained between individuals, with a maximum of 50 people present.

7 Checklist for infection control at schools and after-school programmes

A checklist is given below covering the measures that must be in place before a school or after-school programme can reopen. The measures are described in more detail above. The school owner is responsible for ensuring that infection control measures are in place, and that staff are familiar with the content and implementation of infection control measures[16].

Measure	Date implemented	Remarks
**The school owner's overarching responsibility**		
Train staff regarding infection control measures by familiarising them with the information given in this guide		
Information for parents/guardians concerning new routines at schools/after-school programmes		
Prepare plan for hand washing procedures for pupils and staff		
Prepare written procedure for cleaning		
Prepare plan for establishment and organisation of cohorts		
Establish dialogue with any staff who are in a risk group and children who require special provision		
**Hygiene measures**		
Make sure sufficient soap and paper towels are available at all handwashing stations and toilets		
Training of pupils in handwashing procedures and cough etiquette		
Put up posters about handwashing procedures and cough etiquette		
Provide alcohol-based disinfectants where no handwashing facilities are available		
Plan hand hygiene measures to be applied outside or on excursions (wet wipes and alcohol-based disinfectants)		
**Follow social distancing rules**		
Consider the use of rooms relative to the number of pupils in the cohorts		
Planning for outdoor activities, including staggered times for different cohorts		
Divide outdoor areas so that pupils from different cohorts do not mix insofar as is possible		
Avoid large gatherings of pupils		

[16] See Regulation No. 470 of 27 March 2020 relating to infection control measures, etc. regarding the coronavirus outbreak

Measure	Date implemented	Remarks
Ensure that sufficient stationery and other equipment/materials is available to limit sharing		
Provide a separate desk/chair per pupil with a safe distance between pupils		
Provide a separate seat for each pupil during meals and activities, with a safe distance between pupils		
Ensure appropriate procedures for meals and food served in portions		
Plan to reduce congestion in changing rooms, toilets and on the way in to and out of the premises		
If appropriate, apply markings to floors to ensure safe distances are maintained in areas where congestion may occur		
Plan for alternating times for breaks to limit the number of pupils who are outside at the same time		
Plan for additional adults to be out at break times in order to help pupils maintain a safe distance from each other		
Plan for dispersed places where people can assemble before the start of the school day in order to avoid congestion		
Plan school transport		
Avoid using public transport for out of school trips		
**Cleaning**		
Draw up a cleaning plan, which describes the frequency and methods to be used for the various points. The plan must cover toilets, washbasins, frequently touched objects (door handles, stair banisters, light switches, etc.)		
Draw up a plan for cleaning toys, tablets, etc. Toys and items that cannot be cleaned must be tidied away		
**For staff**		
Limit physical meetings, arrange video conferencing where appropriate		
Maintain social distancing during breaks		
Establish procedures for cleaning shared tablets, computers/keyboards		
Limit use of public transport		

8 References

Unicef, WHO, IFRC. Key Messages and Actions for COVID-19 Prevention and Control in Schools. March 2020

CDC. https://www.cdc.gov/coronavirus/2019-ncov/community/schools-childcare/guidance-for-schools.html

Tiltak på skole- og barnehageområdet under koronautbruddet våren 2020, rapport 03.04.2020 på www.udir.no
